# A Home-Based Educational Intervention Improves Patient Activation Measures and Diabetes Health Indicators among Zuni Indians

**DOI:** 10.1371/journal.pone.0125820

**Published:** 2015-05-08

**Authors:** Vallabh O. Shah, Casey Carroll, Ryan Mals, Donica Ghahate, Jeanette Bobelu, Phillip Sandy, Kathleen Colleran, Ronald Schrader, Thomas Faber, Mark R. Burge

**Affiliations:** 1 School of Medicine, University of New Mexico Health Sciences Center, Albuquerque, New Mexico, United States of America; 2 Indian Health Services Comprehensive Center in Zuni Pueblo, Zuni, New Mexico, United States of America; Weill Cornell Medical College Qatar, QATAR

## Abstract

**Introduction:**

One in three people will be diagnosed with diabetes by 2050, and the proportion will likely be higher among Native Americans. Diabetes control is currently suboptimal in underserved populations despite a plethora of new therapies. Patient empowerment is a key determinant of diabetes control, but such empowerment can be difficult to achieve due to resource limitation and cultural, language and health literacy barriers. We describe a home-based educational intervention using Community Health Representatives (CHRs), leading to improvement in Patient Activation Measures scores and clinical indicators of diabetes control.

**Methods:**

Sixty participants with type 2 diabetes (T2D) completed a baseline evaluation including physical exam, Point of Care (POC) testing, and the Patient Activation Measure (PAM) survey. Participants then underwent a one hour group didactic session led by Community Health Representatives (CHRs) who subsequently carried out monthly home-based educational interventions to encourage healthy lifestyles, including diet, exercise, and alcohol and cigarette avoidance until follow up at 6 months, when clinical phenotyping and the PAM survey were repeated.

**Results:**

PAM scores were increased by at least one level in 35 (58%) participants, while 24 participants who started at higher baseline score did not change. Six months after intervention, mean levels of A1C decreased by 0.7 ± 1.2%; fasting blood glucose decreased by 24.0 ± 38.0 mg/dl; BMI decreased by 1.5 ± 2.1 kg/m2; total cholesterol decreased by 12.0± 28.0 mg/dl; and triglycerides decreased by 52.0 ± 71.0 mg/dl. All of these changes were statistically significant (p<0.05).

**Conclusion:**

This six month, CHR led and community-oriented educational intervention helps inform standards of practice for the management of diabetes, engages diabetic populations in their own care, and reduces health disparities for the underserved population of Zuni Indians.

**Trial Registration:**

ClinicalTrials.gov NCT02339311

## Introduction

The number of American Indians and Alaska Natives (AI/ANs) who have diabetes is growing rapidly, especially among young people. At more than 16%, AI/ANs have the highest age-adjusted prevalence of diabetes among all U.S. racial and ethnic groups [1]. One such population is the Zuni Pueblo home to a small, geographically isolated tribe located in a rural portion of New Mexico, USA. It is home to ~11,000 Zuni Indians and over 90% of all Zunis live in the Pueblo. This socioeconomically disadvantaged population faces a major public health challenge from growing health disparities. Changing lifestyles have led to decreased physical activity and increased caloric intake with high consumption of fast food, soda pop and alcohol. Therefore, the Zuni are experiencing interrelated epidemics of obesity, diabetes, hypertension, kidney disease and intermediate phenotypes.

In the late 1980’s, the prevalence of diabetes in persons aged 35 years and older among Zuni Indians was 28 percent [2], and 32% of individuals aged 20 years and older who were receiving care at the Zuni Indian Health Service (IHS) clinic had diabetes. Diabetes control is currently suboptimal in underserved populations despite a plethora of new therapies. Patient empowerment is a key determinant of diabetes control, but such empowerment can be difficult to achieve due to resource limitation and cultural, language and health literacy barriers. This bourgeoning diabetes crisis requires an aggressive multifactorial approach to treatment and prevention. Patient self-care is a necessary component of glycemic control. Patients need to be involved in all aspects of their care, including decision-making around medical management, healthy eating, weight loss, physical activity, and glucose self-monitoring.

The Chronic Care Model re-orients healthcare practice to promote a systematic, planned approach by emphasizing productive interactions between informed, activated patients, their families, and proactive healthcare teams [3, 4]. A central tenet of this concept is that healthcare workers cannot achieve optimum outcomes when working alone. Rather, desired outcomes are achieved when communities and healthcare organizations work together toward shared goals. The Patient Activation Measure (PAM) is a validated tool that assesses a patient’s ability to effectively participate in their care. It specifically evaluate an individual’s knowledge, skill, and confidence for managing their health and healthcare. Individuals that measure high on this instrument typically understand the importance of taking a proactive role in managing their health and have the skills and confidence to do so. The PAM survey measures patients on a 0–100 scale and can differentiate patients into one of four “activation levels” along an empirically derived continuum. Each activation level reveals insight into an array of health-related characteristics, including attitudes, motivators, behaviors, and outcomes.

Previously, as part of the Zuni Health Initiative (ZHI), we surveyed participants regarding barriers to healthcare [5], with particular attention to diabetes care. In ZHI we also collected and stored clinical phenotype information and anthropological measurements from all participants. Participants identified the following barriers: access to care, language barriers, limited patient education, and anxiety around diagnosis, fear of chronic disease, reluctance to participate in self-care, resistance to dietary change, and reluctance to engage in regular exercise. We have previously documented suboptimal glycemic control with a high burden of kidney disease among the Zuni [6, 7]. The burden presented by these barriers ultimately translates into a lack of patient activation and engagement in their healthcare, effectively hindering adoption of healthy behaviors. Focus groups subsequently identified common solutions to overcome some of these barriers, including home-based care, point of care testing, individualized exercise and nutrition prescriptions, and care providers with knowledge of the Zuni language, community and culture.

The information gathered during these focus groups [8] was used to design and implement a Zuni culture specific educational intervention in diabetes. We devised an innovative educational intervention based on the coordination of four key elements: (a) delivering healthcare that incorporated collaborative communication within the healthcare team and emphasized greater autonomy in care, adherence to the medical regimen, and patient-centered goal setting, all while retaining the ability to address the needs of patients, family members, the healthcare team, and/or the healthcare system; (b) providing innovative educational and organizational approaches, as well as behavior change strategies, that enhanced adherence; (c) addressing health beliefs that reduced adherence by over- or under-predicting maladaptive thoughts (e.g., catastrophizing, minimizing, cognitive dissonance, invincibility, or fatalism) or that interfered with weight control; and (d) using technology to address barriers to achieving desired health outcomes. We report here the results from an intervention conducted by Community Health Representative (CHRs) using group classes and home-based teaching, point of care testing, and individualized exercise and nutritional programs.

## Methods

The protocol for this trial and supporting TREND checklist are available as supporting information; see S1 TREND Checklist and S1 Protocol. This study was approved by the University of New Mexico Health Sciences Center Human Research Review Committee and the Indian Health Service Institutional Review Board, and all participants rendered written informed consent. Study subjects received $25 each time they participated. This observational clinical study was conducted from June 2012 to Dec 2012 and was retrospectively registered at ClinicalTrials.gov (NCT02339311). The study was not initially registered due to the fact that it is an observational design with education as the intervention that did not clearly meet the criteria for inclusion on ClinicalTrials.gov, and registration was not required by our Institutional Review Board. We confirm that all ongoing and related trials for this observational educational intervention are now registered.

### Study Participants

**Fig 1:**
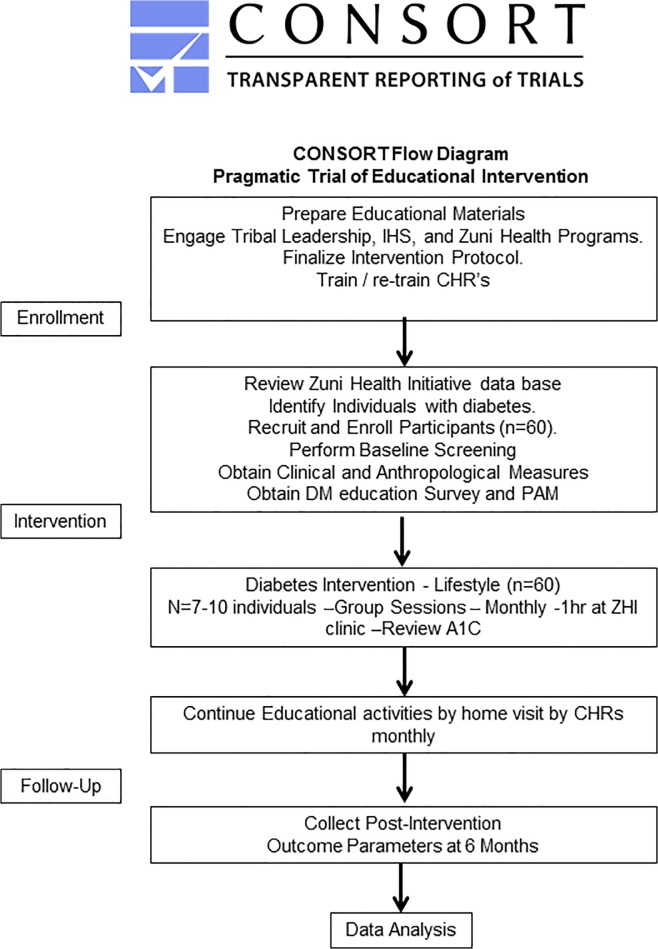
Pragmatic trial of educational intervention—CONSORT Participant Flow Chart – Study Design.

Inclusion criteria incorporated adults aged 18 years or older with a diagnosis of diabetes mellitus. Potential study participants were recruited by Zuni CHRs using the ZHI project database, which contains diabetes status as established by the American Diabetes Association (ADA) within the past 12 months(i.e. fasting plasma glucose >125 mg/dl, A1C > 6.5%, and/or random blood glucose >200 mg/dl with symptoms [9]. CHRs then contacted eligible participants based on A1C > 6.5% randomly by phone or home visit. Additional subjects were recruited using fliers around town and newspaper announcements. CHRs did home visits to complete baseline study measurements, including physical exams, point of care testing and survey completion. All 60 participants with diabetes recruited at baseline remained throughout the six months of pragmatic trial of educational intervention (Fig 1) and there was no dropout from meetings or classes, and there were no missing observations due to the fact that the intervention was home based outreach as an important component of our study design.

### CHRs as Lay Interventionists

Three Zuni community members aged 37–45 with a background in health-related work were recruited, trained and certified as lay interventionists by the University of New Mexico’s Project Extension for Community Healthcare Outcomes (Project ECHO) [10]. They were trained on lifestyle coaching, diabetes prevention, and diet and exercise change. They assisted in teaching lifestyle classes and provided support and guidance to study participants. The CHR staff has also received extensive training in adult education including i) diabetes management, ii) the theoretical framework of intervention, iii) group management skills, and iv) implementing an interventional protocol. The initial training was approximately 100 hours in duration and was performed using telemedicine. Delivery of the intervention was supervised by Vallabh Shah. To monitor intervention fidelity, the home-based educational intervention was codified in the ZHI Manual of Study Operations to assure standardization for all CHR’s with continuous evaluations and was used as the basis for continuing CHR training.

### Lifestyle Classes

Education classes were held at the Zuni Health Initiative building. Each class lasted approximately 60 minutes, with 40 minutes of PowerPoint presentations and 20 minutes for questions and review of A1C results. Class reminders and transportation to and from class was provided. Each class accommodated 7–10 participants. The educational component of the class followed the nutritional and lifestyle modifications for diabetes recommended by the ADA [11], along with discussions about the taboos and stigmata associated with diabetes in Zuni. Small group classroom classes were followed by home-based, individualized education, and POC testing. Participants attended monthly group lifestyle classes during the intervention period. Modified lesson plans from the Diabetes Prevention Program [12] were used as a starting point for developing culturally appropriate lifestyle curricula. During the intervention period, classes focused on diabetes, diabetes prevention, increasing physical activity, decreasing sedentary activities, and making diet and behavior modifications for weight loss and disease prevention. Recommended diet modifications focused on decreasing total calories and fat by avoiding fried foods and sweetened beverages, increasing fiber intake, reducing glycemic load by switching from refined wheat to unrefined wheat flour, and reducing sugar intake. Professional nutritionists and fitness instructors from the Zuni Health Program helped guide dietary modifications and referred to information published by the American Dietetic Association on general nutrition [13] and guidelines on the management of diabetes [9].

Participants were asked to exercise at least 150 minutes per week in addition to their normal activity. The exercise intervention was specifically designed to increase adherence and acceptability by creating activities that were flexible and culturally appropriate. Participants were asked to walk on their own or within walking groups organized by CHR’s at least three times per week. Walking has been shown to be an effective exercise for decreasing diabetes risk [14]. Participants were also encouraged to do alternate forms of aerobic exercise, if they chose, at the ZHI exercise facility. Participants were placed into peer support groups of 4–6 individuals. This “buddy system” has been shown to increase compliance in interventional [15, 16] and cross-sectional studies of exercise participation [17–19].

Didactic classes were conducted by two medical students (CC and RM) at the 6^th^ grade reading level, and these addressed the human body and organ functions, including pancreatic endocrine function and the potential complications of diabetes, including hypertension, dyslipidemia and kidney disease. The importance of medications and lifestyle modification, improving diet, and exercising 150 minutes per week were emphasized. The education team also discussed portion control as a strategy to improve nutrition by presenting simple ways of reducing caloric intake. The classes were also designed to address issues specific to the Zuni culture, including the high rate of kidney disease and diabetes in the Zuni population, and traditional foods and dietary habits that contribute to high caloric intake. Participants had the opportunity to discuss their A1C value with instructors after the class.

Two surveys were completed with the assistance of CHRs. The Educational Intervention Survey is a 40 item survey that includes basic demographic information about the participant as well as diabetes specific questions. A validated Patient Activation Measure (PAM) instrument was also completed by participants using the short form PAM questionnaire [20–26]. The response options for the 13 questions use a categorical agreement scale with 4 response options: (i) strongly disagree, (ii) disagree, (iii) agree, (iv) strongly agree, and N/A. The raw score was calculated by adding responses to the 13 questions. If all questions are answered (i.e., no “N/A” is used), the range of raw scores is 13 to 52. If there is at least 1 item with a response of N/A, the total score will be divided by the number of items completed and multiplied by 13 to yield a normalized raw score. A nomogram provided by Dr. Hibbard [27] under a licensing agreement converts raw scores to an “activation score,” ranging from 0 to 100 with a classification level between 1 and 4: Level 1- Believing the patient’s role is important but not taking action; Level 2- Having the confidence and knowledge necessary to take action; Level 3- Taking action to maintain and improve one’s health; and Level 4- Staying the course even under stress. All study procedures were repeated 6-months later, following the intervention.

### Statistical analysis

#### Study Sample Size

We initially chose the A1C measurement as our outcome variable for the purpose of calculating sample size, and we considered a difference in A1C of 0.3% between PAM Levels to be clinically important, with sigma equal to 0.3. We assumed that baseline A1C levels would approximate 7.9±2.0%, as reported of 3373 American Indians participating in a large, multidisciplinary, intensive case management demonstration project within the Indian Health Service among 138 tribes in 13 states [28]. Since we planned on analyzing many biochemical markers and anthropological measures (such as A1C, fasting lipids, BMI, blood pressure, and PAM scores), we employed the Bonferroni inequality to adjust the Type I error rate to 0.01. With alpha = 0.01 and beta = 0.9, we required 60 participants based on the two-sample t-test and assuming equal variance.

For the purposes of this manuscript, the primary outcome variable was PAM levels. Secondary outcomes included body mass index (BMI), hemoglobin A1C, total cholesterol (TC), and triglycerides (TG). Raw PAM scores (the sum of responses to the 13 questions) were converted to PAM activation scores (range 0–100) according to an algorithm [20]. PAM levels were defined as follows: Level 1 has a PAM activation score of <47.0; Level 2 has a PAM activation score of 47.1–55.1, Level 3 has a PAM activation score of 55.2–67.0, and Level 4 has a PAM activation score of >67.1. For the purposes of a robust retrospective power analysis for the proportion of subjects who will demonstrate a longitudinal improvement in PAM level, and assuming by null hypothesis that 10% of patients will improve, our sample size of 60 is adequate to detect that 27% of subjects will improve with 80% power and alpha = 0.01 using a one-sample binomial test. This is reasonable given that we observed an improvement in PAM level in 58% of subjects.

We used SAS ver. 9.3 and R ver. 3.03 for all analyses [29]. Baseline and six month follow-up values of demographic and clinical characteristics of the diabetes cohort were summarized as Mean (SD) or as Median [Interquartile Range], depending upon the distribution of values. P-values for changes from baseline to six month follow-up were calculated using paired t-tests or paired Wilcoxon signed rank tests, with the latter being employed in the case of extreme skewness or outliers in the distribution of values.

We performed simple regression analyses by determining the association of baseline PAM stage with clinical variables using Spearman correlation with exact p-values to capture the ordinal nature of PAM levels and to adjust for outliers and tied values. We similarly calculated the association of follow-up PAM stage with follow-up clinical variables. Finally we used regression analysis to adjust for gender, baseline clinical variable, and baseline PAM level to examine the effect of baseline PAM level on follow-up clinical values. We used the same method of replacing baseline PAM level with change in PAM from baseline to follow-up to examine the effect of change in PAM score on follow-up clinical value.

## Results

Sixty diabetic participants completed the study. Study demographics are shown in Table 1. The average age of participants at baseline was 49.4±12.0 years and it did not change significantly at six month follow-up (50.1±12.0, P>0.07). The majority of participants were women (68% females, 32% males). As shown in Table 1, pre- and post-intervention values for BMI, A1C, TC, and TG, all showed statistically significant improvements (P<0.05).

**Table 1:** Demographic and clinical characteristics of fasting study participants at baseline and at 6 month of follow-up.

	Baseline Values (n = 60) (Mean ± SD) or Median [Interquartile Range]	Six Months Follow-up Values (n = 60) (Mean ± SD) or Median [Interquartile Range]	P Value
**BMI**	33.8 ± 8.4	32.4 ± 8.2	0.001
**Glucose (mg/dl)**	158.6 ± 69.9	134.8 ±45.5	0.0003
**A1c (%)**	8.12 ± 2.16	7.39 ± 1.6	0.001
**Total Cholesterol (mg/dl)**	159.4 ± 37.1	147.9 ± 31.4	0.003
**Triglyceride (mg/dl)**	161.0 [111.0, 233.5]	123.0 [98.0, 192.0]	0.001
**BUN (mg/dl)** *	14.0 [11.0, 19.0]	14.5 [10.0, 24.0]	0.027
**Creatinine (mg/dl)** *	0.80 [0.7, 1.00]	0.80 [0.7, 1.00]	0.33
**UACR (mg ALB/g CR)** *	34.5 [15.0, 114.9]	52.3 [18.4, 337.2]	0.017
**Uric Acid (mg/dl)**	5.1± 1.8	4.8± 1.6	0.115

* Median [Lower Quartile, Upper Quartile].

**Fig 2:**
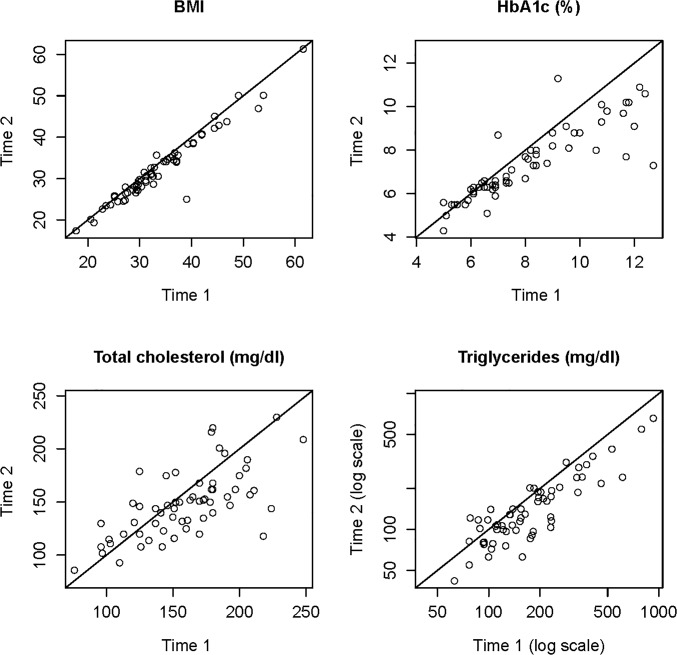
Agreement plots (n = 60). For each clinical parameter, the patient’s value at baseline screening (time 1) is on the horizontal axis and the 6-month follow-up value (time 2) is on the vertical axis. The plotted line is the line of perfect agreement between times 1 and 2. Points below the line represent a decrease from time 1 to time 2, while points above the line represent an increase from time 1 to time 2.

The agreement plots shown in Fig 2 depict the reductions in BMI, A1C, TC, and TG at base-line (time1) vs. 6 months of educational intervention (time 2).

We observed the overall PAM distribution to be significantly changed after six months of educational intervention. At baseline, we found 9 patients (15%) at level 1; 11 patients (18%) at level 2; 19 patients (32%) at level 3, and 21 patients (35%) at level 4 of activation. The distribution of PAM after the six month intervention was 3 patients (5%) in level 1; 0 patients (0%) in level 2; 7 patients (12%) in level 3 and 50 patients (83%) in level 4. Thirty-five participants increased their PAM level by one or more levels, 24 participants remained at the same PAM level, and one patient’s PAM level declined. These findings were statistically significant (P<0.0001).

**Fig 3:**
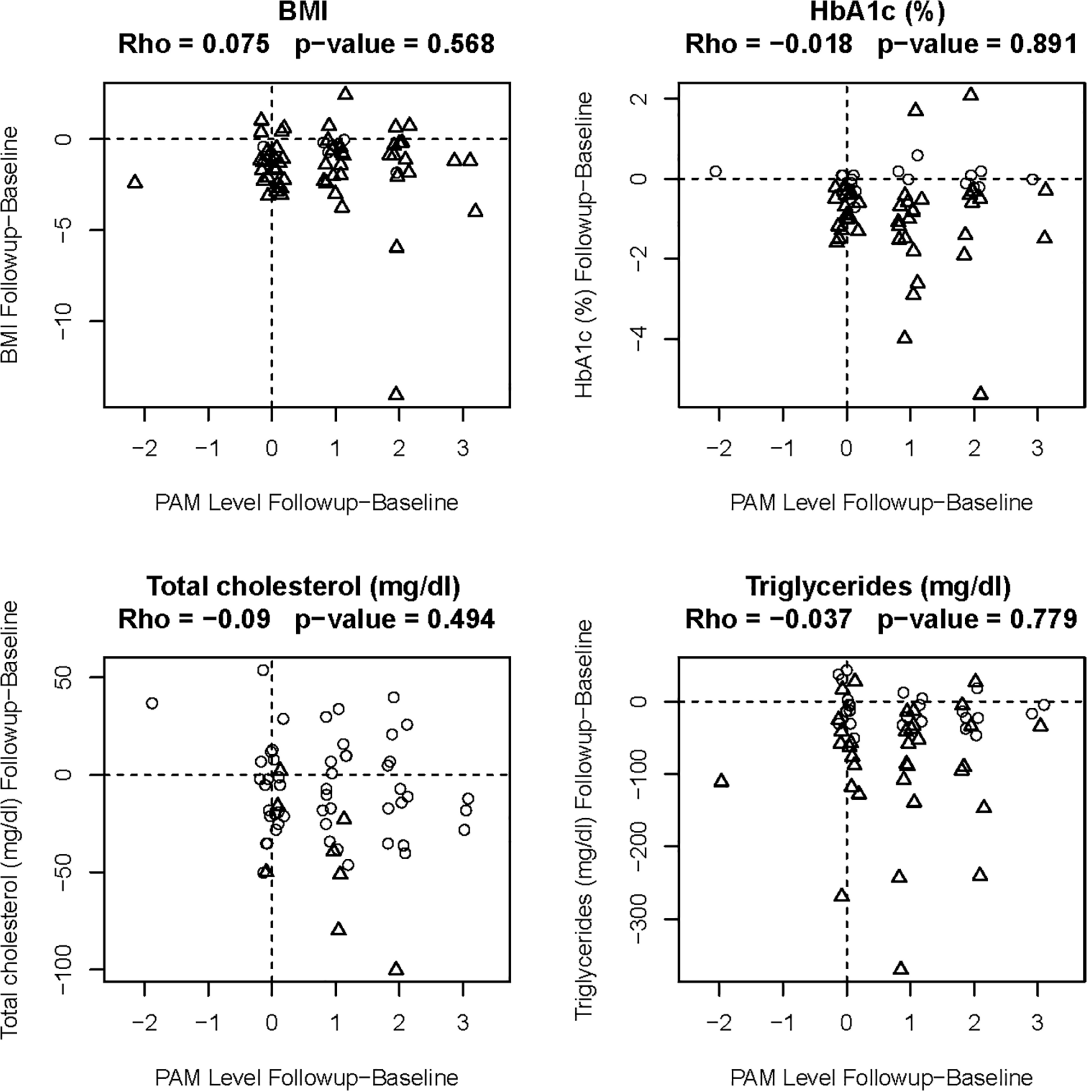
Regression analysis showing changes in clinical parameters vs. changes in PAM levels (n = 60). For each clinical parameter the change in PAM level from baseline to 6 months is depicted on the horizontal axis and change in the clinical parameter from baseline to 6 months is depicted on the vertical axis. Reported values are Spearman’s rho (nonparametric correlation coefficient), and the corresponding p-value (calculated on original, not jittered values). A more sophisticated regression analysis adjusting for age, gender and baseline values had similar p-values and is not shown.

Correlation between PAM levels at baseline screening and changes in clinical parameters from baseline to 6-months follow-up, and correlations between changes in PAM level and changes in clinical parameters from baseline to 6-months follow-up were calculated. Our six month educational intervention demonstrated significant improvement in activation levels (Fig 2), but changes in clinical parameters were not correlated with either PAM level at baseline nor change in PAM level from baseline to 6 months reflecting limitation in our analysis which was not sufficient for a causal inference. PAM level was not a significant predictor of any follow-up clinical value, after adjusting for baseline clinical value (Fig 3), reflecting our distribution of 67% participants in levels 3 and level 4 of activation at the baseline.

## Discussion

Multiple studies show that PAM scores are predictive of health behaviors, including prevention behaviors (e.g. obtaining screenings), healthy behaviors (e.g. healthy diet and regular exercise), self-management behaviors (e.g. monitoring and medication management), and health information seeking [22–25, 30]. Individuals with higher levels of activation have better health outcomes and lower rates of healthcare utilization, such as emergency department use and hospitalization [31, 32]. There is further evidence that it is possible to increase activation levels with education and appropriate intervention [33, 34].

Studies have demonstrated that patients at lower activation levels do not take control of their own health and often lack basic knowledge about their condition, whereas patients with high activation scores tend to possess the knowledge, skills, and confidence to self-manage their disease under adverse circumstances. Patients with higher activation scores are also more likely to exercise on a regular basis, eat a low fat diet with more fruits and vegetables, and abstain from smoking, resulting in better self-reported health and fewer ER visits [35]. A retrospective analysis showed that patients with higher PAM scores had lower A1C levels and lower rates of all-cause hospitalization [36].

We have observed that a home-based, CHR-implemented educational intervention using POC testing and individualized therapeutic goals significantly increases PAM and occurs concomitantly with improvements in metabolic parameters relevant to diabetes, including BMI, A1C, and lipid concentrations. While it is possible that these metabolic improvements are attributable to factors other than the intervention, it seems likely that the home based intervention we employed was responsible for these improvements and that the inability to establish a correlation between improvement in PAM and improvement in metabolic parameters is a by-product of our inclusion of several participants who had high levels of activation (and thereby, self-efficacy) at baseline, as discussed below. The PAM instrument has been validated in several populations, including older adults [30, 37–38]. Various interventions have been shown to increase patient activation and possibly improve outcomes, but previous studies have not shown this increase to translate into improved diabetes control. Mayberry et al. demonstrated that high levels of patient activation correlated with self-management behaviors but not with glycemic control and concluded that in order for PAM to affect glycemic control, the highest level of activation may need to be achieved [35].

In our study, PAM levels increased significantly. After six months, 83% of patients were at stage 4 of activation, compared with 35% at baseline. There are likely to be several reasons PAM scores increased in our study. Using CHRs who were members of the participant community likely increased their knowledge of patient culture, language, resources, and barriers, thereby allowing provision of education in a culturally sensitive manner and at an appropriate level of health literacy. Such CHRs, when appropriately trained, can work individually with patients to tailor care programs to available community resources and overcome barriers through local knowledge. Numerous studies have demonstrated beneficial outcomes from having CHRs as part of the diabetes care team [39–41] and patients report feeling more empowered in their care as a result of CHR interventions.

In our previous study detailing diabetes focus groups, patients reported that access to care, including travel to clinic visits, prolonged wait times, and the lack of a relationship with care providers were major impediments to care. Home-based visits are thus an attractive alternative, offering patient convenience, avoidance of waiting rooms and the sterile atmosphere of clinics, and allowing patients to be in a comfortable and familiar environment during the intervention sessions.

Point of Care testing also offers many benefits. Receiving laboratory results at the time of the visit allows the medical team and the patient to make immediate decisions based upon those test results, including recommended changes to self-care behaviors, medication adjustments, and/or referral to specialist. POC testing has been shown to be accurate as long as the equipment is certified and routinely calibrated, and it is cost effective [42]. In gestational diabetes patients receiving a simple exercise program without behavioral recommendations, POC testing was associated with a reduction in maternal postprandial glucose, A1C, CRP, triglycerides and maternal or neonatal complications, but not in fasting glucose values [43].

It is beyond the scope of the study to determine which individual aspect of the intervention (CHR home visits or POC testing) may have contributed to the beneficial changes observed in PAM. Regardless, we have demonstrated that an innovative, six month, patient-centered intervention can empower patients to become active participants in their care, and such activation can result in improved clinical outcomes. Although improvement was observed in many of the biomarkers of diabetes and metabolic control that we collected among the subjects who participated in our educational intervention, we were unable to relate this improvement to an improvement in the PAM score. This likely reflects the relatively large number of participants who had high PAM scores at baseline, suggesting a high degree of self-efficacy at the outset. Additionally, by forcing the raw PAM scores into four categories, we lost important information, and potentially power, in our regression analysis. Future studies will need to be powered to include only those who can realistically improve their PAM score at baseline. One of our challenges and limitations to this study is the lack of a no-intervention control group. We used a longitudinal study design in T2DM patients with baseline data collection before and after the six month of intervention. This design is appropriate for the demonstration of feasibility and for initial proof of concept, and it is cost effective, provides reliable parameter estimates with small samples, and is pragmatic because it allows for “real-world” variability in variables. The design has informed us that the educational intervention we employed is associated with statistically significant changes in clinically relevant outcomes but these are not necessarily causally related. Aside from these advantages, we chose this particular design because the Zuni culture is somewhat resistant to testing interventions with no-intervention control groups. Another limitation of the study is that it was initially powered to observe a change in A1C rather than PAM scores.

Based on our progress to date and knowledge gained, diabetes prevention in real-world settings should continue to be a major focus among health-disparate Native American groups [44]. In conclusion, a home-based, CHR-driven educational intervention led to significant improvements in patient activation and biochemical parameters of diabetes control, as well as risk factors associated with diabetes, in this cohort of Zuni Natives with diabetes.

## Supporting Information

S1 TREND Checklist TREND Checklist. (PDF)

S1 Protocol Evaluating a Novel Approach to Diabetes Management in Zuni Indians. (PDF)
