# Supplementary material for: A Home-Based Educational Intervention Improves Patient Activation Measures and Diabetes Health Indicators among Zuni Indians
Source: PLoS One. 2015 May 8;10(5):e0125820. doi: 10.1371/journal.pone.0125820 (PMC4425648; doi:10.1371/journal.pone.0125820)

Evaluating a Novel Approach to Diabetes Management in Zuni Indians

Research Protocol

June – Dec 2012

Abstract

Purpose: One in three people will be diagnosed with diabetes by 2050, and the proportion will likely be higher among Native Americans. Diabetes control is currently suboptimal in underserved populations such as Zuni Indians despite a plethora of new therapies. Patient empowerment is a key determinant of diabetes control, but such empowerment can be difficult to achieve due to resource limitation and cultural, language and health literacy barriers. We will conduct a home-based chronic disease (diabetes) educational intervention using Community Health Representatives (CHRs) to associate improvement in Patient Activation Measures scores (primary outcome) and clinical indicators of diabetes control (secondary outcomes).

Condition	Intervention
The study focus is on lifestyle changes and patient Activation in Diabetic Patients by regular monthly visit by the CHRs	Educational intervention of Lifestyle and patient activation.

Study type: Observational

Study Design: Cohort, Prospective

Biospecimens Retention: NONE

Primary Outcome Measure:

- Patient Activation (time -6 months of education) (No safety issue)
  The Patient Activation Measure (PAM) instrument completed by participants using the short form PAM questionnaire. The response options for the 13 questions use a categorical agreement scale with 4 response options: (i) strongly disagree, (ii) disagree, (iii) agree, (iv) strongly agree, and N/A. The raw score is calculated by adding responses to the 13 questions. If all questions are answered (i.e., no “N/A” is used), the range of raw scores would be 13 to 52. If there is at least 1 item with a response of N/A, the total score will be divided by the number of items completed and multiplied by 13 to yield a normalized raw score. All study procedures were repeated 6-months later, following the intervention.

Secondary Outcome Measures:

- Diabetes associated health indicators including HbA1c, Glucose, Lipids, BMI (time -6 months of education) (No safety issue)

Enrollment: 60 (both men and women)

Study start date: June 2012

Study completion date: December 2012

Number of arms: 1

Eligibility: All participants are Zuni Natives living in Zuni Pueblo, New Mexico. They are all diabetics with HbA1c level of 6.6% and above in the age range of 21 years to 75 years old.

Sampling Method: Non-probability samples recruited directly from the list of previous study conducted at Zuni.

Summary Description: The number of American Indians and Alaska Natives (AI/ANs) who have diabetes is growing rapidly, especially among young people. At more than 16%, AI/ANs have the highest age-adjusted prevalence of diabetes among all U.S. racial and ethnic groups. One such population is the Zuni Pueblo home to a small, geographically isolated tribe located in a rural portion of New Mexico, USA. It is home to ~11,000 Zuni Indians and over 90% of all Zunis live in the Pueblo. This socioeconomically disadvantaged population faces a major public health challenge from growing health disparities. Changing lifestyles have led to decreased physical activity and increased caloric intake with high consumption of fast food, soda pop and alcohol. Therefore, the Zuni are experiencing interrelated epidemics of obesity, diabetes, hypertension, kidney disease and intermediate phenotypes.

As part of the Zuni Health Initiative (ZHI), we surveyed participants regarding barriers to healthcare, with particular attention to diabetes care. In ZHI we also collected and stored clinical phenotype information and anthropological measurements from all participants. Participants identified the following barriers: access to care, language barriers, limited patient education, and anxiety around diagnosis, fear of chronic disease, reluctance to participate in self-care, resistance to dietary change, and reluctance to engage in regular exercise. We have previously documented suboptimal glycemic control with a high burden of kidney disease among the Zuni. The burden presented by these barriers ultimately translates into a lack of patient activation and engagement in their healthcare, effectively hindering adoption of healthy behaviors. Focus groups subsequently identified common solutions to overcome some of these barriers, including home-based care, point of care testing, individualized exercise and nutrition prescriptions, and care providers with knowledge of the Zuni language, community and culture.

The information gathered during these focus groups is used to design and implement a Zuni culture specific educational intervention in diabetes. We have devised an innovative educational intervention based on the coordination of four key elements: (a) delivering healthcare that incorporated collaborative communication within the healthcare team and emphasized greater autonomy in care, adherence to the medical regimen, and patient-centered goal setting, all while retaining the ability to address the needs of patients, family members, the healthcare team, and/or the healthcare system; (b) providing innovative educational and organizational approaches, as well as behavior change strategies, that enhanced adherence; (c) addressing health beliefs that reduced adherence by over- or under-predicting maladaptive thoughts (e.g., catastrophizing, minimizing, cognitive dissonance, invincibility, or fatalism) or that interfered with weight control; and (d) using technology to address barriers to achieving desired health outcomes.

Sixty participants with type 2 diabetes (T2D) completed a baseline evaluation including physical exam, Point of Care (POC) testing, and the Patient Activation Measure (PAM) survey. Participants then underwent a one hour group didactic session led by Community Health Representatives (CHRs) who subsequently carried out monthly home-based educational interventions to encourage healthy lifestyles, including diet, exercise, and alcohol and cigarette avoidance until follow up at 6 months, when clinical phenotyping and the PAM survey were repeated

Background and Rationale

Obesity and Diabetes: A Global Epidemic

Obesity, a 21st-century medical scourge to which many providers have become desensitized, currently afflicts over 300 million individuals worldwide.[1] While 68% of the American population is considered overweight, our prevalence of overt obesity has skyrocketed to 30% since 1970.[2] Despite being an inherently pathophysiological state, obesity often translates into a higher risk for severe cardiovascular disease such as hyperlipidemia, myocardial infarction, stroke, hypertension, and type 2 diabetes mellitus (T2DM). In turn, worldwide obesity-related healthcare costs are estimated to exceed $215 billion—and continue to rise exponentially.[3]

Similarly, type II diabetes exacts a steep toll on the American medico-economic climate. Based on the National Health and Nutrition Examination Survey (NHANES) in 2007, the estimated prevalence of diabetes in America surpasses 7.8% of the general population and represents over 23.6 million people. Almost 1 in 5 Native Americans with primary providers from Indian Health Services are diabetic. Resulting medical costs from downstream medical complications such as coronary heart disease, stroke, nephropathy, neuropathy, and dialysis treatment exceed $174 billion.[4]

Unfortunately, Native American populations bear a disproportionate burden of both obesity and diabetes, compared to all other US populations; 40.1% of American Indian men are obese, in contrast with African-American (26.5%), Hispanic (26.6%), and Asian (2.7%) men.[5] Roughly 16.5% of the total American Indian population has been diagnosed with diabetes, compared to American non-

Hispanic white (6.5%), Asian-American (7.5%), Hispanic (10.4%), and African-American (11.8%) populations.[6]

Zuni Indian Population

Situated in rural, western New Mexico, the Zuni Pueblo is comprised of approximately 11,000 Zuni Indians. Largely endogamous, Zunis tend to value and cultivate multigenerational families, reinforced by low emigration; only 20% live outside the pueblo. Most pueblo members derive income through home-based crafts (e.g. jewelry, fetishes), but the pueblo's socioeconomic status is progressively deteriorating. In turn, most pueblo residents face enormous health care disparities. Moreover, lifestyle shifts during the past century have involved declining physical activity, and increasing intake of high-calorie foods.

Given this socioeconomic and behavioral climate, it comes as no surprise that the Zuni now suffer from a "perfect storm" including a high prevalence of obesity, diabetes, kidney disease, hypertension, and dyslipidemia. Historically, interventions for high-risk populations like the Zunis have been relatively unsuccessful; confounding effects of cultural, historical, and economic barriers not only undermine clinical compliance, but also discourage utilization of available health care services.[5,6]

Health Disparities Among Zuni Indians

Within the healthcare context, disparity usually refers to the experience of adverse health outcomes by patients who are considered minorities. Health care disparity occurs among a broad backdrop of socioeconomic inequality, but can also be exacerbated by internal factors within the health care delivery system.[7,8,9]

Tragically, American Indians (particularly Zuni's) have a lower health status when contrasted with nearly all other U.S. racial or ethnic populations; they experience a disproportionate disease burden, underutilize healthcare services, and their average life expectancy falls 2.4 years short of the

national average. An IHS report identifies low education, high poverty, health care discrimination, and cultural factors as culprits.

Chronic disease hits harder in Zuni. IHS data reveal a mortality rate for obesity and T2DM 2.9 times higher in American Indians, versus the American population. Further health disparity exists in addressing metabolic syndrome, chronic kidney disease, and cardiovascular disease. Whereas Native Americans' chronic disease-related mortality is high, their disproportionate medical burden increasingly burdens the IHS budget and raises healthcare costs.[10]

Disparities in Chronic Diseases Among American Indians

As proposed by the International Diabetes Federation in 2006, "metabolic syndrome" refers to a chronic condition characterized by comorbid metabolic risk factors for T2DM and cardiovascular disease; namely, increased waist circumference, as well as any pair of the following: 1) high serum triglyceride levels (≥ 150 mg/dL); 2) low HDL cholesterol (< 50 mg/dL in women, < 40 mg/dL in men); 3) high blood pressure (> 130 mmHg systolic, or >85 mmHg diastolic, or undergoing treatment for hypertension); 4) increased free plasma glucose (or history of T2DM). The "medicalization" of this symptom constellation underscores its severity. Once identified, patients must undergo dramatic lifestyle modification focusing on weight reduction and physical activity; otherwise, they are at high relative risk for developing type 2 diabetes, cardiovascular disease, or other associated conditions such as chronic kidney disease, polycystic ovary syndrome, fatty liver disease, or hepatocarcinoma.[11]

In 2008, the population-based, longitudinal Strong Heart Study determined that metabolic syndrome afflicted 38% of all American Indians in the United States. This study further demonstrated that obesity rates in American Indians (AI/AN) exceed the national average.[12] Overweight and obesity in the AI/AN population exceeds the rates for all other U.S. ethnic groups combined.[5] Perhaps most frightening is the trend of increasing overweight and obesity in not only adults, but school-aged

children as well; AI/AN children are at 104% greater risk for being overweight versus non-AI/AN counterparts.[13,14]

Likewise, T2DM affects the U.S. AI/AN population tremendously. CDC data confirms an over-two-fold relative prevalence of T2DM in AI/AN adult versus the national average.[15] This statistic continues to climb; in the decade preceding 2004, T2DM prevalence in AI/ANs more than doubled.[16] In particular, AI/AN children have experienced a marked rise in T2DM prevalence. Among Pima Indians, whose T2DM rates are documented as the highest world-wide, saw a near-doubling in diagnosis of children from 1990 to 1998.[17] Naturally, the rising number of AI/AN diabetics has led to an upsurge of diabetic complications within Native American communities.

Contributing Factors to Health Disparities

Diet

Historically, unemployment and poverty among AI/ANs has engendered a reliance on federal commodity programs, but these programs often fall short of nutritional requirements.[18] With a paucity of healthy options in tribal grocery stores, Tribal-residing AI-ANs often fall prey to ever-expanding "fast-food" chains around reservations, ingesting diets that are high-calorie, high-fat, and high-sugar. Navajo Reservation grocery stores routinely offer produce in poor condition, and rarely sell low-fat products. Furthermore, the population's limited demand of perishable produce has dis-incentivized grocers from selling it at an affordable price, and in reasonable volume.[5]

Socioeconomic Factors

Regardless of region, all low-income and ethnic minority American populations with a high prevalence of obesity are more likely to have economic stress, scarcer venues for exercising, less access to reasonably-priced healthy food, and saturation by advertising of calorie-rich foods.[18] Recent

consensus suggests that, in America, disparities in overweight/obese prevalence occur primarily because of varying socioeconomic status.[19]

The Native American socioeconomic environment is characterized by poverty and unemployment in the greater part of its communities. AI/AN poverty rates were over twice the general populations' in 1999. A study in 2000 illustrated that adult AI/AN males earned $8200 less than the national male average; likewise, adult AI/AN women fell short by $4400.[20]

The Native American community's debilitating socioeconomic condition greatly detracts from its members' living conditions, diet, employment status, physical activity level, and overall health.

Psychosocial

Research by Whitbeck et al. describes a "soul wound" among AI/AN populations; cross-generational historical trauma and grief layers over one's life span, negatively affecting emotional health and mental well-being. Largely, this trauma is attributed to the centuries of adversity AI/ANs have experienced in America. This adversity clearly persists, manifested as poor economic status, sense of cultural disconnection, and discrimination.[21]

Additionally, no evidence yet suggests that the AI/AN cultural framework views obesity as a medical problem. Often, when over half of an AI/AN population experiences overweight/obesity, members adopt a normalization and resignation towards the issue.[5] More so, a 1997 study concluded that Navajo adults over the age of 60 perceived moderate overweight to be healthy.[22]

Physical Activity

A 2005 National Institues of Health (NIH) study disclosed that AI/AN adults are roughly 13% less likely to perform physical activity, compared to their White and Asian counterparts.[23] Similarly, both the Strong Heart Study and Navajo Health and Nutrition survey revealed decreased levels of physical activity in reservation-residing AI/ANs between 45 and 74 years old. Specifically,

researchers identified safety concerns, lack of motivation/willpower, lack of time, and limited child care as barriers to activity.[24,25]

Reducing Health Disparities Among American Indians

Considering AI/ANs' exponentially-growing health disparities, developing more effective approaches to address public health and health care delivery in their communities is crucial. Current consensus points toward community-based strategies as a worthwhile starting point for such efforts; these strategies would emphasize tribal liaisons, and respect tribal culture.[15]

Community-Based Strategies

The positive effect of community-based lifestyle interventions for preventing chronic disease is well-documented. In the 1990's, research on community-based risk reduction projects showed that community based activities, such as exercise classes and cholesterol screenings, could increase the level of physical activity prevalence rate for screening tests in a community, all on a relatively modest budget.[26] Previous community-based interventions in Native American populations in New Mexico have been effective at reducing weight and improving glycemic control when compared to a control group.[27] Community-based interventions can also be cost effective. A study in the Netherlands found that intervention costs needed to prevent 1 new case of diabetes were 2,000-9,000 Euros for community-based interventions compared to 5,000-21,000 Euro for standard healthcare interventions.[28,29] Successful programs in the past have emphasized cultural relevance, self-efficacy, and a reliance on the community-researcher relationship. Thus, any intervention targeting chronic diseases like obesity and diabetes within the Zuni community must be congruent with their traditional culture and values. In devising a procedure, input from tribal community members and leaders must be sourced, particularly due to their instrumental role in promoting lifestyle change.[30,31]

Identifying Cultural Barriers to Health Care in Zuni

Several barriers have been identified by the U.S. Commission on Civil Rights which play a role in NA/NI health disparities. These include discrimination, ethnic bias, racism, a paucity of culturally-competent care, and adverse environmental factors.[27] Zuni Health Initiative (ZHI) interviews have demonstrated additional barriers. Structural barriers involve: 1) cost and inconvenience of transportation to/from doctors' appointments; 2) the transient nature of the communities healthcare provider workforce; 3) perception of misdiagnosis or late diagnosis of disease; 4) perception of health care rationing; 5) language barriers; 6) poor patient education. Psychological barriers comprise: 1) anxiety; 2) fear of diagnosis of chronic disease; 3) reluctance to practice self-care; 4) resistance to dietary modification; and, 5) resistance to periodic physical activity.

Rationale

This research project aims to conduct an observational, qualitative and quantitative study, to evaluate the use of a novel chronic disease prevention approach for improving clinical outcomes and health-related quality of life in Zuni Indians with chronic disease. Our approach will attempt to address the factors leading to health disparities, as described in *Background.* Specifically, this multifaceted approach will assess the efficacy of a culturally sensitive, home-based diabetes prevention (HBDP) model, while determining which of the populations' perceptions are most prohibitive to chronic disease management. Zuni participants will have undergone prior baseline metabolic profile testing, completed a Patient Activation Measure (PAM) survey to determine patient engagement, and answered Educational Intervention Survey to gather more data on lifestyle factors. Essentially, our role is to hold periodic, informal classes for all participants about lifestyle modification measures, and diabetes pathophysiology. During these classes, we will measure participants' Hemoglobin A_{1C} values, and provide each attendee an opportunity to deliberate over the results with us after class.

Along the same time frame, employees of the Zuni Health Initiative will perform lab work (for comparison of metabolic profiles to baseline values), and the PAM survey (for comparison to initial response). Finally, a three-month follow-up will take place, including both quantitative (blood/urine analysis, survey) values and qualitative (survey). Ultimately, this study will provide a highly-relevant assessment of the effectiveness of a community-based, culturally-sensitive HBDP model in addressing chronic disease.

Methods

The protocol for this proposal has IRB approval both **from UNM Health Sciences Center IRB # 10249 and Indian Health Services IRB # N10-AQ-10**. Both IHS and the tribal council of Zuni have approved this research. All participants of this pilot education program must be diagnosed with overt diabetes (fasting glucose >126mg/dL, HgA1c>6.5%, or random blood glucose >200mg/dL). The goal is to recruit Zuni adults to attend one group class no larger than 10 individuals. Participants are recruited by native Zuni Community Healthcare Representatives (CHRs) as part of the larger Zuni Health Initiative (ZHI) project. Dr. Shah and the ZHI have extensive experience recruiting community members as part of the Zuni Kidney Project (ZKP). The CHRs will contact diabetics identified during the ZKP for recruitment into this study, as well as advertise with fliers around town and in the local newspaper.

As individuals are recruited to the study, baseline measurements taken by the CHR in the home of the participant. Baseline lab work, including a complete lipid panel, CBC, UACR, and serum creatinine collected from each participant at home prior to the class. Clinical measurements such as height, weight, blood pressure and HgA1c is performed on site the day of the class. Point of care (POC) testing is done with HgA1c levels, has the advantage of giving the patient immediate feedback on lab results. A prospective, randomized, controlled study in Australia showed that measuring cholesterol using point of care testing, along with education about lifestyle changes during home visits, led to a statistically significant decrease in cholesterol levels in the interventional group, whereas no change was reported in the control group.[39] In our study, the individual results of the HgA1c is given to each participant at the end of the class, allowing each participant the opportunity to discuss his or her results with the class instructor and to develop personalized strategies for lower HgA1c levels through lifestyle modifications and adherence to a treatment plan.

The Educational Intervention Survey is completed by the participant during the home visit prior to the pilot class. The Educational Intervention Survey is an 8 page survey that included basic demographic information about the participant as well as diabetes specific questions. The diabetes specific questions allow the participants to self-report strategies they use to manage their diabetes and complications they have faced as diabetics.

A Baseline Patient Activation Measure (PAM) score is obtained prior to the pilot class using the 13-item short form PAM questionnaire. The 13 questions use a Likert scale grading with 4 response options: Strongly Disagree (1), Disagree (2), Agree (3), Strongly Agree (4), and N/A.[40,41] The raw score is then converted to an activation score and the patient is placed in 1 of 4 stages: 1. Believing the patient's role is important but not taking action, 2. Having the confidence and knowledge necessary to take action, 3. Taking action to maintain and improve one's health, 4. Staying the course even under stress.[42] Patients at lower activation levels do not take control of their own health and lack basic knowledge about their condition, whereas patients with high activation scores possess the knowledge, skills, and confidence to self-manage and stay the course under adverse circumstances. Patients with higher activation scores are more likely to exercise on a regular basis, eat a low fat diet with more fruits and vegetables, and abstain smoke, which result in self-reported better health and lower rates of doctor office visits, ER visits, and hospital stays.4 A retrospective analysis showed that patients with higher PAM scores had better A1C testing results, better A1C control, and lower rates of all-cause hospital discharges.[43] Because higher PAM scores are correlated to better patient outcomes, it is an ideal way to measure if educational classes improve participants' knowledge and perception about diabetes. After our education courses, we hypothesize the participants' PAM scores will increase.

The education class is held at the Zuni Health Initiative building, located across the street from IHS. Each class run for approximately 60 minutes, with 40 minutes of Powerpoint presentations and 20 minutes for questions and interpretation of individual A1C results. Each class consist of 6-10 participants. The classes begin at noon and lunch is provided. The participants are reminded of the class 48-72 hours beforehand, and CHR provide transportation for those who need it.

The educational component of the class follow the nutritional and lifestyle modifications for diabetics recommended by the American Diabetes Association.[44] The class cover the pathophysiology, classification, and the potential complications of diabetes including hypertension, dyslipidemia and kidney disease. The importance of medications and lifestyle modifications, improving diet and exercising 150 minutes per week, for management of diabetes is discussed. We presented portion control as a strategy to improve nutrition by presenting simple ideas participants can use to cut calories. The classes are designed to be interactive, and the participants are encouraged to ask questions during or after the presentation. The classes are also designed to address issues specific to the Zuni culture, including the high rate of kidney disease and type 2 DM in the Zuni population as well as traditional foods and dietary habits that contribute to high caloric intake. Participants are also encouraged to write questions on a note card that are collected at the end of the Powerpoint, so questions can be asked anonymously. Each participant got the opportunity to discuss their A1C value with the instructors after the class.

We believe a Student's t distribution is most closely represent our data. We anticipated that a 2-tailed statistical model is most appropriate. We found that the standard deviation for Hemoglobin A1C would be approximately 0.5% with a mean of 5.0 in normal subjects. We assume that most clinicians will consider a difference in Hemoglobin A1c of 0.3% to be clinically important.[39] We wish to limit the Type I error rate to 0.05 or less and to achieve a power of at least 90% (i.e. a Type II error rate of 0.1). We chose the Hemoglobin A1C measurement to model this sample size estimation. Since we are planning on evaluating a total of 8 biochemical markers and survey instruments (HgA1C, lipid panel, BMI, BP, and PAM scores) we employed the Bonferroni inequality to adjust the Type I error rate. Under these assumptions, we calculate that approximately 60 participants are required. We proposed to collect an additional 6 participants in case non-parametric analysis seems appropriate.

CHR made home visits once a month to check on the participants' progress and reinforce the ideas presented in the classes. Six months after the class, the baseline lab work and clinical measurements repeated. Participants also repeated the PAM questionnaire. We hypothesize that the participants' HgA1C, lipid panel (total cholesterol, HDL, LDL, triglycerides), BMI, BP, and PAM scores will have improved from their baseline numbers taken before the education course and follow up with a CHR.

Possible Difficulties

One potential difficulty will be recruiting enough participants to participate in the classes. We need 60 participants for a statistical analysis from the relatively small community. We plan to reach our recruitment goal by advertising the classes to the community through radio announcements and fliers, as well as contacting past participants from the ZKP that qualify for inclusion in this study.

Language may also be a potential barrier in this study. The classes will be conducted in English. Approximately 90% of Zuni's speak a language other than English at home.[45] Although most

of the people of Zuni know English, some of the elderly may not be fluent in English or may prefer to communicate in their native tongue. For this reason, local CHR will be present at all of the classes and will be available to translate to any participant that is not comfortable speaking in English.

Limitations and Biases

One limitation of our study may be its focus on diabetes, versus other illnesses. However, we believe that by developing a targeted approach as described within the small, indigenous Zuni population will be highly scalable for future efforts against other chronic conditions.

Furthermore, we cannot account for patient attrition that may result over the course of our study. With a relatively small cohort, it is crucial that we maintain a high retention rate for the various aspects of the study, in order to generate statistically-significant results.

One of the strengths of this study is its singular, culturally-sensitive approach to the Zuni people. Presumably, this may limit our future ability to translate the curriculum for other populations.

References

1. World Health Organization. *Global Strategy on Diet, Physical Activity and Health*. *World Health Organization*. 2012. <http://www.who.int/dietphysicalactivity/strategy/eb11344/strategy_english_web.pdf>.
2. Flegal, K. M., M. D. Carroll, and C. L. Ogden. "Prevalence and Trends in Obesity Among US Adults." *JAMA* 303.3 (2010): 235-41. Print.
3. Hammond, R. A., and R. Levine. "The Economic Impact of Obesity in the United States." *Diabetes, Metabolic Syndrome and Obesity: Targets and Therapy* 3 (2010): 285-95. *Dovepress*. Web. 26 Oct. 2012. <http://www.dovepress.com/the-economic-impact-of-obesity-in-the-united-states-peer-reviewed-article-DMSO-recommendation1>.
4. *National Diabetes Fact Sheet: General Information and National Estimates on Diabetes in the United States, 2007.* Vol. 2007. Atlanta: Centers for Disease Control and Prevention, 2007. Print.
5. Halpern, Peggy. "Obesity and American Indians/Alaska Natives." U.S. Department of Health and Human Services, 2007. Web. 26 Oct. 2012. <http://aspe.hhs.gov/hsp/07/AI-AN-obesity>.
6. Stidley, C. A., V. O. Shah, and M. Scavini. "The Zuni Kidney Project: A Collaborative Approach to an Epidemic of Kidney Disease." *Journal of the American Society of Nephrology* 14.90002 (2003): 139S-43. Print.
7. Jha, A. "Differences in Medical Care and Disease Outcomes among Black and White Women with Heart Disease." *ACC Current Journal Review* 13.1 (2004): 22. Print.
8. Philbin, E. F., P. A. McCullough, T. G. DiSalvo, G. W. Dec, P. L. Jenkins, and M. D. Weaver. "Underuse of Invasive Procedures among Medicaid Patients with Acute Myocardial Infarction." Circulation, 2003. Web. 26 Oct. 2012.

9. Powe, N. R. "To Have and Have Not: Health and Health Care Disparities in Chronic Kidney Disease." *Kidney International* 64 (2003): 763-772. *Nature*. Web. 26 Oct. 2012. <http://www.nature.com/ki/journal/v64/n2/full/4493945a.html>.
10. United States. U.S. Department of Health and Human Services. Indian Health Service. *The Indian Health Service Fact Sheets*. N.p., Jan. 2006. Web. 28 Oct. 2012. <http://www.ihs.gov/PublicAffairs/IHSBrochure/>.
11. Meigs, James B. "The metabolic syndrome (insulin resistance syndrome or syndrome X)." UpToDate. Ed. Jean E. Mulder, David M. Nathan, and Joseph I. Wolfsdorf. N.p., 6 Oct. 2011. Web. 26 Oct. 2012.
12. Welty, T. "Health Implications of Obesity in American Indians and Alaska Natives." *American Journal of Clinical Nutrition* 53 (1991): 1616S-20S. Print.
13. Lucove, J., S. Vupputuri, G. Heiss, K. North, and M. Russell. "Metabolic Syndrome and the Development of CKD in American Indians." *American Journal of Kidney Disease*. N.p., Jan. 2008. Web. 29 Oct. 2012. <http://www.ncbi.nlm.nih.gov/pubmed/18155529>.
14. "Pediatric Nutrition Surveillance System (PedNSS)." *Centers for Disease Control*. N.p., 2005. Web. 26 Oct. 2012. <http://www.cdc.gov/pedness/what_is/pednss>.
15. "Measuring Healthy Days- Population Assessment of Health Related Quality of Life." *CDC and US Department of Health and Human Services*. 1-44, 2000. Web. 26 Oct. 2012.
16. Acton, K. J., and N. R. Burrows. "Diagnosed Diabetes Among American Indians and Alaska Natives Aged." *Centers for Disease Control and Prevention*. N.p., n.d. Web. 29 Oct. 2012. <http://www.cdc.gov/mmwr/preview/mmwrhtml/mm5544a4.htm>.
17. Acton, K. J., N. Rios Burrows, K. Moore, L. Querec, L. S. Geiss, and M. M. Engelgau. "Trends in Diabetes Prevalence Among American Indian and Alaska Native Children, Adolescents, and Young Adults." *American Journal of Public Health* 92.9 (2002): 1485-490. Print.

18. Kumanyika, Shiriki Kinika, and Sonya Grier. "Targeting Interventions for Ethnic Minority and Low-Income Populations." *The Future of Children* 16.1 (2006): 187-207. Print.
19. "The Surgeon General's Call to Action to Prevent and Decrease Overweight and Obesity." *Office of the Surgeon General*. DHHS/Public Health Service, n.d. Web. 26 Oct. 2012. <http://www.surgeongeneral.gov/library/calls/obesity/index.html>.
20. Ogunwole, Stella U. *We the People: American Indians and Alaska Natives in the United States*. Washington DC: n.p., 2006. Print.
21. Whitbeck, Les B., Gary W. Adams, Dan R. Hoyt, and Xiaojin Chen. "Conceptualizing and Measuring Historical Trauma Among American Indian People." *American Journal of Community Psychology* 33.3/4 (2004): 119-30. Print.
22. White, L. L., C. Ballew, T. J. Gilbert, J. M. Mendlein, A. H. Mokdad, and K. F. Strauss. "Weight, Body Image, and Weight Control Practices of Navajo Indians: Findings from the Navajo Health and Nutrition Survey." *Journal of Nutrition* 10th ser. 127 (1997): 2094-098. Print.
23. Barnes, P. M., P. F. Adams, and E. Powell-Griner. "Health Characteristics of the American Indian or Alaska Native Adult Population." *National Health Statistics Reports* 20 (2010): 1-24. Print.
24. Mendlein, J. M., D. S. Freedman, and D. G. Peter. "Risk Factors for Coronary Heart Disease among Navajo Indians." *Journal of Nutrition* 10th ser. 127 (1997): 2099. Print.
25. Yurgalevitch, Susan M., Andrea M. Kriska, Thomas K. Welty, Oscar Go, David C. Robbins, and Barbara V. Howard. "Physical Activity and Lipids and Lipoproteins in American Indians Ages 45-74." *Medicine & Science in Sports & Exercise* 30.4 (1998): 543-49. Print.
26. Brownson RC et al, Preventing cardiovascular disease through community-based risk reduction: the Bootheel Heart Health Project. AJPH 86: 206-113, 1996.

27. Gilliland SS et al, Strong in Body and Spirit: Lifestyle intervention for Native American adults with diabetes in New Mexico. Diabetes care 25: 78-83, 2002.
28. Jacobs-van der Bruggen M et al, Lifestyle Interventions Are Cost-Effective in People With Different Levels of Diabetes Risk : Results from a modeling study. Diabetes care 30: 128-134, 2006.
29. Gaziano TA and Reddy S, Scaling up interventions for chronic disease prevention: the evidence. Lancet 370: 1939-46, 2007.
30. Charles-Azure J, and E. Little. Promotion of Healthier Beverages in Indian Communities. *The IHS Primary Care Provider*. 30:6, 2003.
31. Satterfield DW et al, Community-Based Lifestyle Interventions to Prevent Type 2 Diabetes. Diabetes care 26: 2634-52, 2003.
32. U.S. Commission on Civil Rights (2004). *Broken Promises: Evaluating the Native American Health Care System.* Washington, DC: Retrieved from *<http://www.usccr.gov/pubs/nahealth/nabroken.pdf>*
33. Bantle JP, Wylie-Rosett J, Albright A, et al. Nutrition recommendations and interventions for diabetes. Diabetes Care. 2008; 31(1):S61-S78. dio: 10.1097/JAC.0b013e3181ba6e77.
34. Dabelea, D., R. L. Hanson, and P. H. Bennett. "Increasing Prevalence of Type II Diabetes in American Indian Children." *Diabetologia*. N.p., 1998. Web. 26 Oct. 2012. <http://www.ncbi.nlm.nih.gov/pubmed/9726592>.
35. Hibbard JH, Stockard J, Mahoney ER, Tusler M. Development of the Patient Activation Measure (PAM): Conceptualizing and Measuring Activation in Patients and Consumers. Health Serv Res. 2004;39(4): 1005-1026. dio: 10.1111/j.1475-6773.2004.00269.x.
36. Hibbard JH, Mahoney ER, Stockard J, Tusler M. Development and testing of a short form of the patient activation measure. Health Serv Res. 2005;40(6):1918 -1930. dio: 10.1111/j.1475-6773.2005.00438.x.

37. Harnack, L., M. Story, and B. Rock. "Diet and Physical Activity Patterns of Lakota Indian Adults." *Journal of the American Dietetic Association* 99.7 (1999): 829-35. Print.

38. Patient Activation Measure (PAM) License Package. Portland, OR: Insignia Health; 2007.

39. Peterson GM, Fitzmaurice KD, Nauton M, et al. Impact of pharmacist-conducted home visits on the outcomes of lipid-lowering drug therapy. J Clin Pharm Ther. 2004;29(1): 23-30. doi: 10.1046/j.1365-2710.2003.00532.x.

40. Remmers C, Hibbard J, Mosen DM, Wagenfield M, Hoye Re, Jones C. Is patient activation associated with future health outcomes and healthcare utilization among patients with diabetes? J Ambul Care Manage. 2009; 32(4):320-327. dio: 10.1097/JAC.0b013e3181ba6e77

41. Salbe, A. D., C. Weyer, and R. S. Lindsay. "Assessing Risk Factors for Obesity between Childhood and Adolescence: Birth Weight, Childhood Adiposity, Parental Obesity, Insulin and Leptin." *Pediatrics* 110 (2002): 299-306. Web.

42. Satterfield, D. W., M. Volansky, C. J. Caspersen, M. M. Engelgau, B. A. Bowman, E. W. Gregg, L. S. Geiss, G. M. Hosey, J. May, and F. Vinicor. "Community-Based Lifestyle Interventions to Prevent Type 2 Diabetes." *Diabetes Care* 26.9 (2003): 2643-652. Print.

43. State and county quickfacts: Zuni Pueblo CPD,NM. US Census Bureau. http://quickfacts.census.gov/qfd/states/35/3586595.html. Published September 18,2010. Accessed October 21, 2012.

44. Peterson GM, Fitzmaurice KD, Nauton M, et al. Impact of pharmacist-conducted home visits on the outcomes of lipid-lowering drug therapy. J Clin Pharm Ther. 2004;29(1): 23-30. doi: 10.1046/j.1365-2710.2003.00532.x.

45. Hibbard JH, Mahoney ER, Stockard J, Tusler M. Development and testing of a short form of the patient activation measure. Health Serv Res. 2005;40(6):1918 -1930. dio: 10.1111/j.1475-6773.2005.00438.x.

46. Patient Activation Measure (PAM) License Package. Portland, OR: Insignia Health; 2007.

47. Hibbard JH, Stockard J, Mahoney ER, Tusler M. Development of the Patient Activation Measure (PAM): Conceptualizing and Measuring Activation in Patients and Consumers. Health Serv Res. 2004;39(4): 1005-1026. dio: 10.1111/j.1475-6773.2004.00269.x.

48. Remmers C, Hibbard J, Mosen DM, Wagenfield M, Hoye Re, Jones C. Is patient activation associated with future health outcomes and healthcare utilization among patients with diabetes? J Ambul Care Manage. 2009; 32(4):320-327. dio: 10.1097/JAC.0b013e3181ba6e77

49. Bantle JP, Wylie-Rosett J, Albright A, et al. Nutrition recommendations and interventions for diabetes. Diabetes Care. 2008; 31(1):S61-S78. dio: 10.1097/JAC.0b013e3181ba6e77.

50. State and county quickfacts: Zuni Pueblo CPD,NM. US Census Bureau. http://quickfacts.census.gov/qfd/states/35/3586595.html. Published September 18,2010. Accessed October 21, 2012.

Pragmatic Trial of Educational Intervention

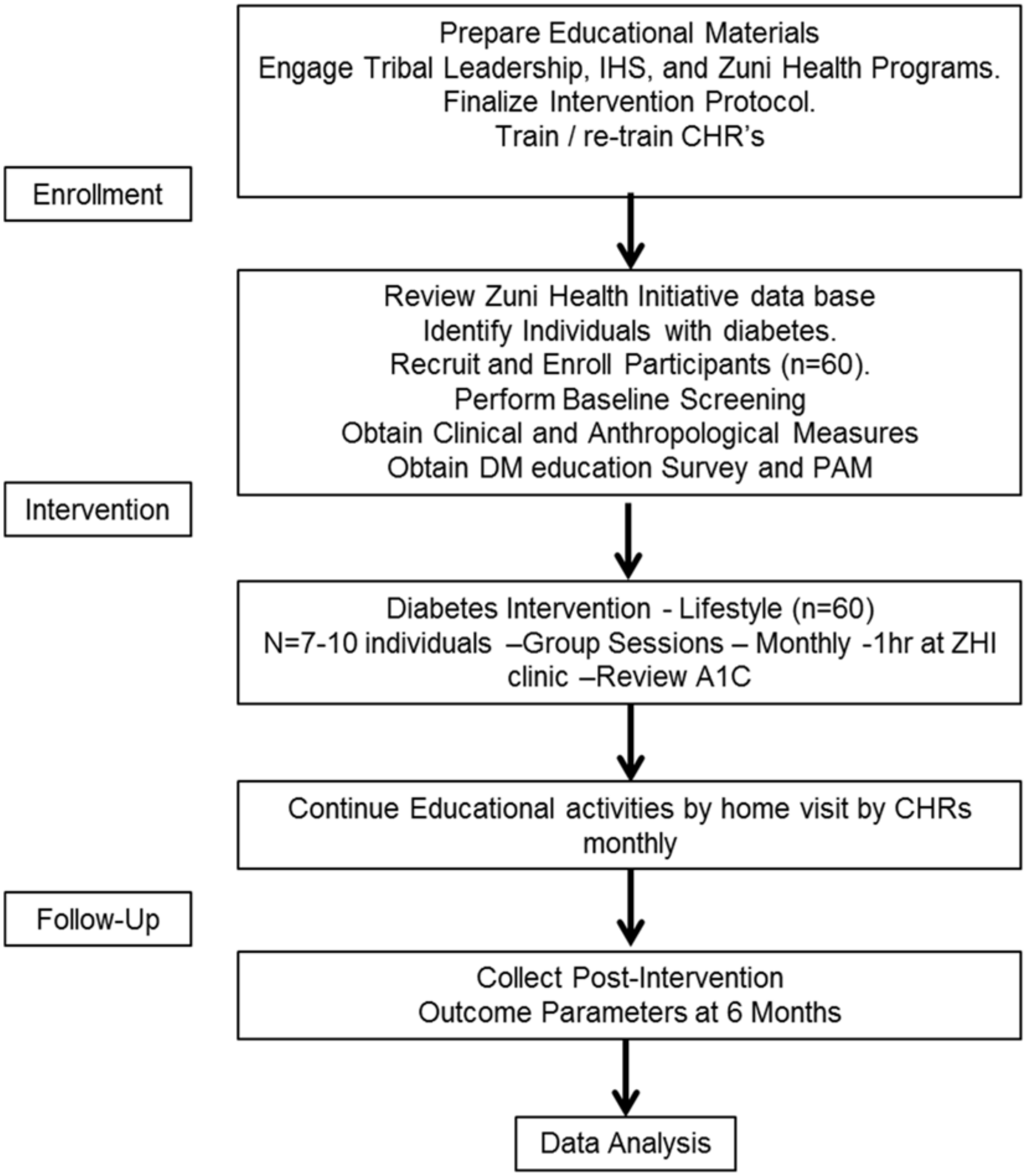

Supplement: S1 Protocol — (PDF) [file pone.0125820.s002.pdf]
